# Concomitant Two-Temporal Cilioretinal Artery Occlusion (CLRAO) With Impending Central Retinal Vein Occlusion (CRVO) in a Young Adolescent With Protein C Deficiency: A Rare Case

**DOI:** 10.7759/cureus.60615

**Published:** 2024-05-19

**Authors:** Pallavi Yelne, Swapneel Mathurkar, Sunil Kumar

**Affiliations:** 1 Medicine, Datta Meghe Institute of Medical Sciences, Wardha, IND; 2 Ophthalmology, Datta Meghe Institute of Medical Sciences, Wardha, IND; 3 Medicine, Jawaharlal Nehru Medical College, Wardha, IND

**Keywords:** cilioretinal artery occlusion, protein c deficiency, anticoagulants, thromboembolism, impending central retinal vein occlusion (crvo)

## Abstract

We report structural changes in the retina of an adolescent diagnosed with the concomitant two temporal cilioretinal artery occlusion (CLRAO) with impending central retinal vein occlusion (CRVO) along with mild protein C deficiency. An 18-year-old girl came to the emergency room with sudden onset painless loss of vision in her right eye. On comprehensive ophthalmic examination, she had a pale superior-temporal retina with spongy macular edema corresponding to two temporal CLRAO and blurred disc margins with mild disc swelling and mild tortuosity of retinal veins all over the retina with few superficial hemorrhages in the right eye corresponding to impending CRVO. Optimal coherence tomography (OCT) showed thickening of the nerve fiber layer in the superior-temporal quadrant involving some part of the macula in the right eye. Perimetry showed a right eye visual field defect in the inferior nasal quadrant. Her coagulation profile was normal but her autoimmune profile was suggestive of mild protein C deficiency. Immediately she was started on anticoagulants. After one month, her visual acuity improved from finger counting close to face to 6/9 with treatment. Over a period of one month, retinal and OCT changes recovered with the same perimetry findings as earlier. This case shows how prompt treatment resulted in dramatic improvement in the form of good visual outcomes.

## Introduction

If neglected, retinal vascular disease can produce sudden, painless blindness with disastrous consequences. Risk factors for central retinal vein occlusion (CRVO) include advancing age, diabetes mellitus, hypertension, being overweight or obese, smoking, and hypercoagulable ailments. It was first described by Osterhuis in 1968 [1]. An example of vascular disease of the retina is CRVO coupled with cilioretinal artery occlusion (CLRAO). The cilioretinal artery (CLRA) has its origin from either the choroidal vascular system or posterior ciliary artery (PCA), which always lacks autoregulation, in contrast to the central retinal artery, which has its origin directly from the ophthalmic artery and has sufficient autoregulation to optimize retinal circulation when there is decreased perfusion pressure. One quadrant, a portion of the macula, half of the retina, or infrequently the whole retina may be supplied by the CLRA. The prevalence of CLRA is 6.9% to 49.5% [2]. Three distinct forms of CLRAO have been identified: two mixed forms that coexist with anterior ischemic optic neuropathy and CRVO, and one isolated form. It is extremely rare for more than one CLRA to occlude at the same time in the same eye [3]. Because protein C performs as an anticoagulant, a lack of it causes a procoagulant state, which enhances the risk for venous thrombosis. Arterial thrombosis is one of the other manifestations of protein C deficiency. However, there is a lack of data even when there are increased chances of arterial thrombosis [4]. In terms of illness severity, protein C deficiency can be congenital or acquired. The prevalence of moderate type cases is believed to be between 1/200 and 1/500, whereas severe clinical cases are extremely rare. In extreme instances, this protein deficiency manifests in neonates' fulminant purpura or disseminated intravascular coagulation (DIC); in moderate protein C deficiency, no symptoms are manifested till adolescence and even if present, typically manifests as recurrent thromboembolism. The majority of moderate and heterozygous instances are asymptomatic, while recurrent thrombosis may be present. The purpose of this rare case report is to provide an overview of the clinical characteristics and excellent visual outcomes achieved by treating a concurrently developing CRVO with two temporal CLRAO in a patient who has a mild protein C deficiency.

## Case presentation

**Figure 1:**
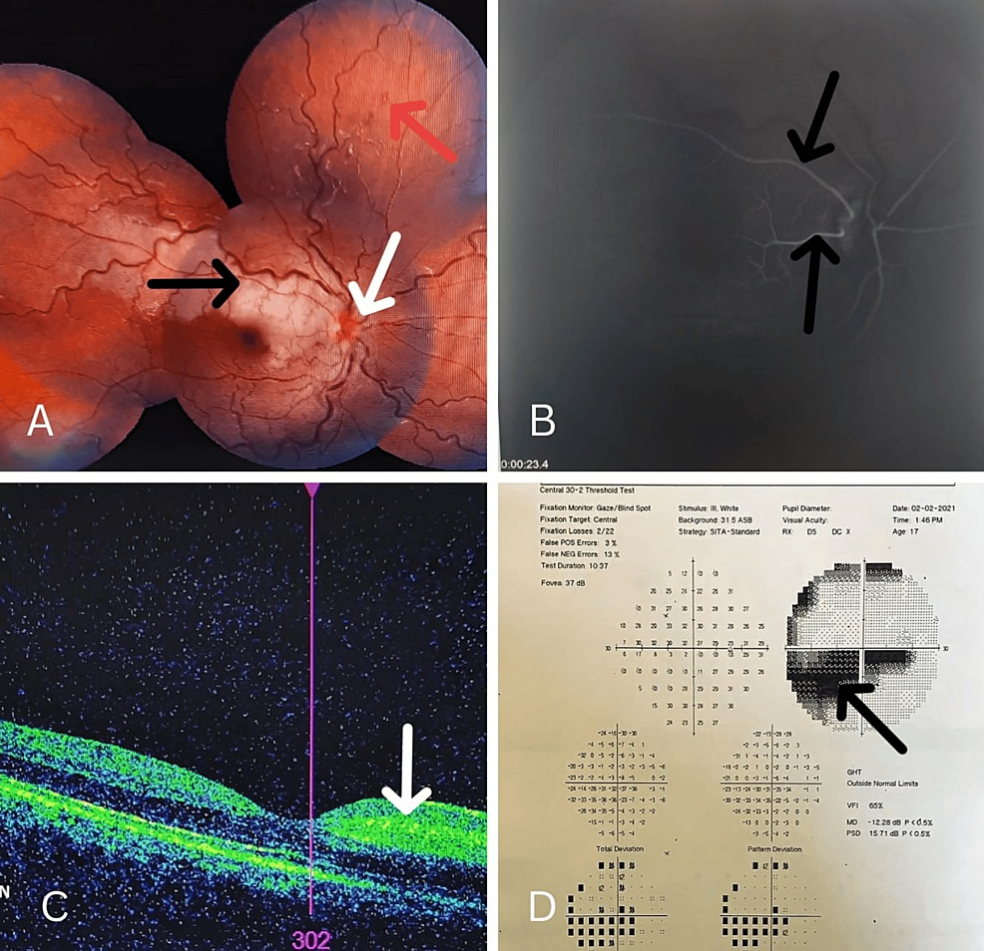
A) Fundus photo right eye, B) FFA, C) OCT, and D) perimetry A) Fundus photo right eye: a pale superior-temporal retina with spongy macular edema corresponding to two temporal CLRAO (black arrow) and blurred disc margins with mild disc swelling (white arrow), and tortuosity of retinal veins all over the retina with few superficial hemorrhages (red arrow). B) FFA showed delayed hyperfluorescence of two temporal CLRAs at 23 seconds (two black arrows) with delayed venous filling (arterial hyperfluorescence at 23 seconds). C) OCT imaging was done that revealed increased nerve fiber layer thickness (white arrow) in the superior-temporal quadrant of the retina involving the superior-nasal part of the macula. D) Perimetry revealed scotoma in the field corresponding to the area having retinal findings (in the inferior-nasal quadrant) with centrocecal scotoma (black arrow). FFA, fundus fluorescein angiography; OCT, optimal coherence tomography; CLRA, cilioretinal artery; CLRAO, cilioretinal artery occlusion

An 18-year-old adolescent girl was presented to an emergency room with sudden severe painless loss of vision in her right eye for about more than 24 hours. She had no associated history of trauma, systemic illness, infection, or any malignancy. Her general and systemic examinations were within normal limits. Her comprehensive ocular examination revealed the best corrected visual acuity (BCVA) of finger counting close to the face in the right eye and normal vision (6/6) in the left eye. There was a relative afferent pupillary defect in the right eye and no other significant anterior segment finding. Both eyes had normal intra-ocular pressure so a dilated fundus examination was done. On fundus examination (Figure 1A), the right eye revealed a pale superior-temporal retina with spongy macular edema corresponding to two temporal CLRAO (black arrow) and blurred disc margins with mild disc swelling (white arrow) and tortuosity of retinal veins all over the retina with few superficial hemorrhages (red arrow) in right eye corresponding to impending CRVO. Fundus fluorescein angiography (FFA) (Figure 1B) showed delayed hyperfluorescence of two temporal CLRAs at 23 seconds (two black arrows) with delayed venous filling (arterial hyperfluorescence at 23 seconds) and few small block-fluorescence over the retina. Optimal coherence tomography (OCT) (Figure 1C) imaging was done that revealed increased nerve fiber layer thickness (white arrow) in the superior-temporal quadrant of the retina involving the superior-nasal part of the macula. Perimetry (Figure 1D) was done that revealed scotoma in the field corresponding to the area having retinal findings (in the inferior-nasal quadrant) with centrocecal scotoma (black arrow).

Detailed workup to identify the etiology of arterial and venous thrombosis was carried out including complete blood count with hemoglobin of 11.2gm/dL, total leucocyte count of 5600/cu mm, and platelets of 240000/cu mm. She had random blood sugar of 112 mg/dL, normal liver function test and normal renal function test with erythrocyte sedimentation rate (ESR) of 12 (raised), homocysteine of 5 umol/L (normal), D-dimer of 40 ng/mL (normal), CRP of 12 mg/dL, which is raised; her coagulation profile was normal including APTT (activated partial thromboplastin time) and PT (prothrombin time). Her cardiovascular evaluation was done. Echocardiography and carotid artery Doppler study were within normal limits. Till this time no risk factor was found. So, we further investigated her and an autoimmune profile was sent. Protein C levels were reduced to 60%, suggestive of mild protein C deficiency. The rest of the autoimmune workups were negative (Table 1).

**Table 1:** Auto-immune profile of patient

Sr. no	Test name and procedure name	Patient value	Control value	Interpretation
1	Lupus anticoagulant: electromechanical clot detector method	42.40 sec	37.20 sec	No lupus-like anticoagulant present
2	Antithrombin III activity: chromogenic method	91%	80%-120%	Normal
3	Protein C functional (chromogenic method)	60%	70%-140%	Mild protein C deficiency
4.	Protein S (free) antigen (immuno-turbidimetry)	80%	60%- 140%	Normal

**Figure 2:**
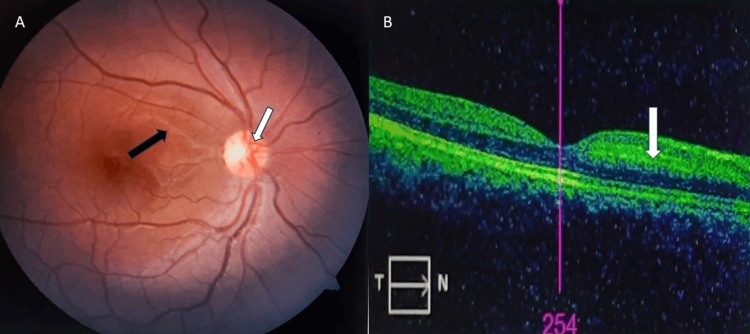
After one month of follow-up. A) Fundus photo and B) OCT A) Fundus photo: retinal whitening in the superior-temporal part of the retina was resolved (black arrow), tortuosity of veins was reduced, and disc edema and hemorrhages were resolved (white arrow). B) OCT showed improvement with a decrease in the thickness of the nerve fiber layer (white arrow). OCT, optical coherence tomography

**Figure 3:**
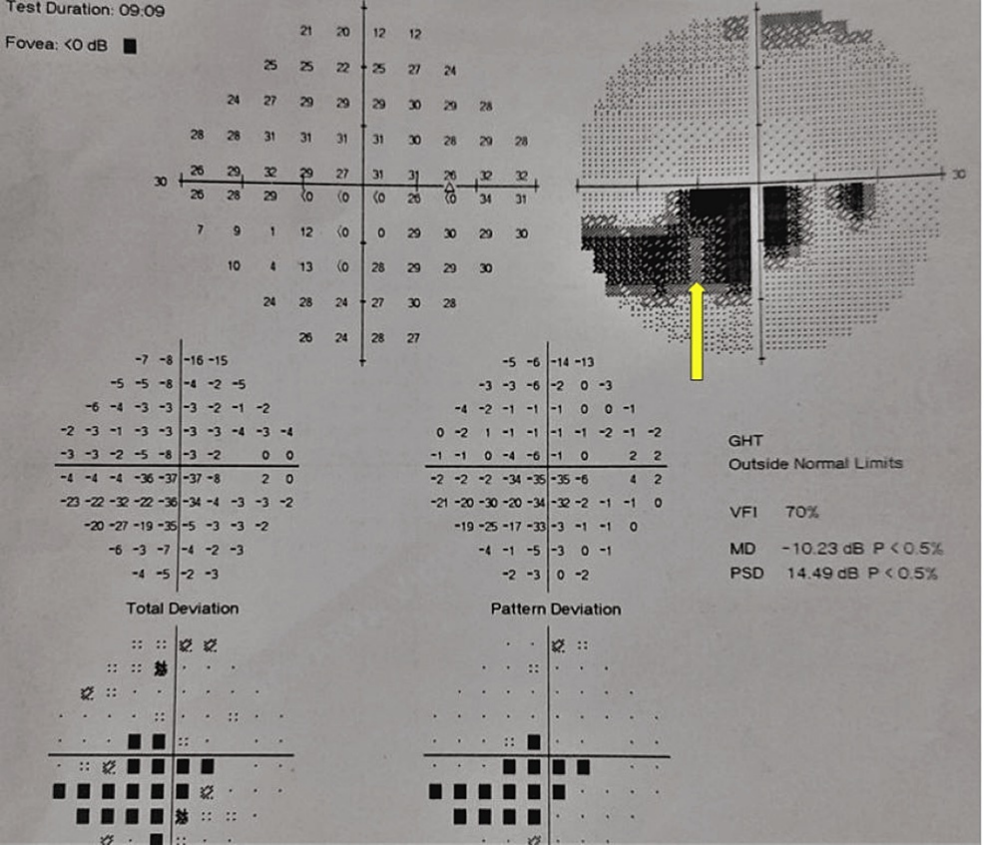
Perimetry showing a slight decrease in scotoma (yellow arrow)

To rule out congenital causes of hypercoagulable state, we investigated Factor V Leiden mutation, prothrombin gene mutation, and MTHFR (methylene-tetra-hydro-folate reductase) gene mutation, which all were negative. Soon after the presentation, the patient was treated with ocular massage, sublingual Isosorbide-dinitrate, tablet acetazolamide 250 mg BD for three days, and topical anti-glaucoma drugs. All medications were given to vasodilate the retinal blood vessels so that blood flow to the retina would be restored. However, when she was diagnosed with protein C deficiency, she was started on systemic anticoagulants, injection fractionated heparin 5000 IU three times a day for five days followed by warfarin 3 mg once a day so that her International normalized ratio would be maintained between 2 and 3. On follow-up after one month, she had an excellent visual outcome. Her retinal findings subsided significantly including retinal whitening in the superior-temporal part of the retina resolved, the tortuosity of veins reduced, and disc edema and hemorrhages resolved (Figure 2A). OCT showed improvement with a decrease in the thickness of the nerve fiber layer (Figure 2B) but perimetry showed a slight decrease in scotoma in one month (Figure 3).

## Discussion

Individuals with protein C deficiency heterozygous form have a later onset of manifestations and a higher thromboembolism risk at an earlier age [5]. Our patient most likely has heterozygous protein C deficiency because she initially showed symptoms at the age of 18. Protein C deficiency can be of two types. The most prevalent type is type 1, which results in a decrease in both the functional activity and concentration of protein C. Only the functional activity has declined in type 2 while the concentration is normal. A number of conditions, including acute thrombosis, medications such as warfarin and oral contraceptives, and liver diseases can also result in protein C deficiency. A patient with protein C deficiency had mixed CRAO and CRVO, according to a case report by Desai et al. [6]. A case study involving 38 patients with CRVO and CLRAO from 1974 to 1999 was described by Hayreh. Compared to cases of CRVO alone, which increased with age, the majority of documented cases of non-ischemic CRVO with CLRAO were young adults [7]. At the first visit, almost all of the patients showed disc edema with hemorrhages, and during the acute phase, all of the eyes had varying degrees of venous engorgement. Usually, FFA does not exhibit the usual CLRAO; instead, it merely exhibits a brief hemodynamic block. In non-ischemic CRVO, the resolution time of retinopathy was mostly eight months in the absence of intervention. There is debate regarding the pathophysiology of CRVO when coupled with CLRA blockage (CLRAO). However, two theories have been proposed [8,9]. First, the elevated capillary pressure brought on by CRVO is the secondary cause of CLRAO, and second, the primary fall in perfusion pressure of the cilioretinal and retinal arteries causes a decrease in retinal circulation, which in turn causes venous stasis and thrombosis. CRVO can occur in conjunction with either branch or central retinal artery occlusion, with the latter accounting for the majority of events [10,11]. About 32% of eyes have CLRAs, which originate through the posterior ciliary circulation. They are mostly dispersed temporally, with just a few distributed nasally. They act as an extra blood supply to the retina. Young adults with combined CRVO and CLRAO generally have a fair prognosis because of the hemodynamic blockage that is transient in nature and the formation of collateral circulation, which frequently helps to restore damage [11]. It was unusual in our case to have two blocked CLRAs on the temporal portion of the optic disc in conjunction with an impending central retinal vein blockage. Furthermore, there has never been a case of CRVO in conjunction with occlusion of the CLRAs due to mild protein C deficiency, making this a rare variety. After receiving treatment as soon as possible, our patient showed improvement in visual acuity, reaching 6/9 in just one month.

## Conclusions

The underlying risk factors for retinal vascular diseases in younger people differ from those in older people. This is probably the first case report of two temporal CLRAs obstruction with impending CRVO caused by even mild protein C deficiency, which had a good visual outcome with systemic anticoagulation therapy. When younger patients have sudden vision loss, a detailed investigation is always necessary to diagnose thromboembolism. An untreated risk factor might result in serious cardiovascular and cerebrovascular incidents. In such cases, to avoid potentially fatal systemic consequences and recurrent thromboembolism, the patient may require long-term anticoagulants.
